# New York Journalism

**Published:** 1879-10

**Authors:** Ausburn Towner


### NEW YORK JOURNALISM.

BY AUSBURN TOWNER.

Every trade or business is as much a sealed book to a person not initiated into its mysteries as is Masonry itself. Given a man a whole and well equipped carpenter shop and he couldn't get out a door, without spoiling a good many feet of lumber; given a man a grocery well stocked and he'd lose money constantly until he learned how to run it; given a man as well appointed a printing office as the Elmira Advertiser, and he'd find his headache a good many times before he could publish a good newspaper and earn anything on the money invested. And, although it is the opinion of the average man that he can edit a journal as well as the next man, and better than half of them are edited, it is the most difficult of all kinds of business to learn thoroughly and succeed at. There used to be a glamour, a sort of a mystery an ill-defined romance connected with newspaper life. With it were suggested an existence of gypsy-like carelessness, ragged clothes to-day, broadcloth of the finest to-morrow, short clay pipes, broad brimmed hats and more or less whiskey.[1] It was on the confines, if not in the centre of a charming Bohemia that ad-

1—More, rather than less.

dressed the sense of most young men with a peculiar attractiveness. Locally, an example of old-time newspaper men could be found in him whilom called "Sparks," better known as such than by his family name of Burdick, or, and he has come down to us from a former generation, the Editor of the Mid-day Sun.[2] These things are so no longer.[3] Journalism is a business now, from the Editor-in-chief, to the littlest newsboy that cries the papers through the streets; as much of a business as selling dry goods, or retailing groceries, and requiring more hard-headed sense than the most complicated commercial operations. It is not genius that is required in this line to-day, it is tact, judgment, plodding industry and quick perceptions. Edgar A. Poe or Lord Byron would be as much out of place on a great morning newspaper of the present time, as either would in a pulpit. A reporter of twenty or twenty-five years ago, such as George Arnold, Fitz James O'Brien, or Mortimer Thompson Doesticks, would stand but a slim chance these days. There are one or two of them left. They do brilliant work now and then when they have an opportunity, but they seldom get it.[4] No man who cannot be trusted to be and keep perfectly sober under the most alluring and enticing circumstances, will hold his position for a minute on any leading New York newspaper. The journalist or reporter of to-day, with but few exceptions, is a gentleman in character, dress and deportment, and not one of them but is entitled to as much consideration as would be given the confidential secretary of the English Chancellor of the Exchequer, and more.

The newspaper man of to-day is a gentleman also, in the broadest acceptation of that term, by education. The notion that the mystery of the business first opened up to a beginner as he stood before the case picking up type, went out with the other mistaken and old-fashioned notion to which reference has been made above, and to-day, the responsible positions in the newspaper offices are so largely as to be almost entirely held by college-bred men.[5] Journalism, from this fact, stands to-day where it does, next to the ministerial, the most important of the professions.

The detail of the work of a large daily is an entirely unknown land to 999 out of every 1,000 men who nevertheless see the result of it every morning of their lives. Let us look at it a minute. In the first place, an Editor seldom, if ever, writes a line for his paper. He is a suggester, a proposer, an architect who makes plans for others to build on. The Editor-in-Chief, the Managing Editor, the City Editor and sometimes the Night Editor are continually busy seeking subjects, either of the character of news opinions, or that require treatment, and they assign men to work up what they may deem of value or interest. The reporter or writer gathers his facts as he may, no matter how far he may have to go for them or how much time he may spend in verifying them, the office, of course, bearing all of the expenses, and writes out his copy. It goes then into the hands of the Night Editor, or one of his assistants, who edits it, puts a head or headings to it and it is then set in type.[6] The Night Editor is the last man who sees it, before it goes in the form and uses his judgment as to its revision, correction or entire rejection.

Some of the newspapers are very particular as to their copy, and have long lists of words or expressions that must not be used, either because of the whim or caprice of the chief, or because of the supposed bad English involved. The public do not know that the leading newspapers are edited with the care and precision that mark the conduct of a quarterly magazine, and it will be a long time before they find it out. So purely an ephemeral thing as a daily newspaper is poor ground on which to work out problems in grammar or the construction of sentences. Not one reader in a hundred thousand, who is interested in some item of news in his paper, knows or cares if the preposition "at" is used incorrectly for "in" or if "learned" is used, when the word should have been "ascertained." Yet two or three, possibly four of the leading newspapers in New York make closer distinctions than those and woe be to the Sub-Editor who lets them slip.

The method of getting news as above shown, where each journal must keep a large staff of reporters to send for it on its own account, is

2—A wonderfully apt name for a journal printed at noon.

3—I speak of New York, not Chicago Journalism.

4—I might name Caleb Dunn whose bits of poetry in the weeklies are immensely superior to the rubbish that finds a place in the monthly magazines, and Thad. McAlpine.

5—As an example: In one of the leading New York morning newspaper offices, there are 55 men in the Editorial Department. Of these, 35 are college graduates, and they occupy all of the most responsible positions. Only 11 of the whole number are printers by trade and 4 of these are College men.

6—Composition or type setting, one of the heaviest expenses of a country daily, is but lightly regarded in some of the New York journals. In the Sun, for instance, they sometimes "kill" as much matter on the galleys as goes into the forms.

changing. There is an Association, that makes it a business to gather the news of the city, manifolds it and furnishes it to whoever will buy, at cheap rates. It reduces greatly the expenses of a newspaper. So that, as far as the mere news of the day is concerned, an Editor can buy it as he would a keg of nails or a pound of soap. This must, eventually, dispose of very many of the reporters attached to the several newspapers, and far from the time coming that Macauleys will write up accounts of murders and descriptions of fire for the morning papers, as Mr. Whitelaw Reid set forth in his advertisement of the Tribune delivered before the New York State Press Association in Rochester last June, that kind of work will be done by the merest scrubs and shoemakers. The eagerness, the almost feverish anxiety for the latest news[7] and especially for exclusive intelligence,[8] a sense[9] quite as much as that possessed by an artist or poet, which it is impossible to acquire and which marks a thorough newspaper man, will, of course, be the means of retaining on all first-class journals some reporters who are to be relied on, but the staffs will, doubtless, be greatly reduced by these associations of which I have spoken.

All the newspapers in New York are conducted on the same general plan as I have already noted, but the different ways of doing the same thing are as marked in this profession as in any other. Every man has his favorite newspaper, inclined towards it, perhaps in the beginning, by its political bearing; he has read it probably for years and thinks its way is the only way, but it may be curious to know the opinions entertained in the profession, of the several members of the profession, as it is sometimes curious to know what ministers may think of other ministers, or lawyers of other lawyers.

It is virtually conceded that in regard to most of the elements that go to make up a great newspaper, the Sun stands head and shoulders above all the rest.[10] Its chief, Charles A. Dana, whatever may be said of his vindictiveness and bitterness, by long service and original capability, has certainly no equal living in the profession. His First Lieutenant, John Swinton, a little erratic on socialistic questions, that he, however, never obtrudes before the readers of the Sun, is a newspaper man born; Ballard Smith, the managing Editor, is without question, the brightest journalist of the younger generation, and John Bogart, the City Editor, is without equal in the position he holds. But with all of this ability at present, the Sun could hardly have become what it is, had it not had at first when under its present control, Amos Cummings for its Managing Editor. And it certainly would not be where it is, had it not been for Dr. John B. Wood, the Night Editor who came there when Mr. Dana did, and who now, for nearly eleven years, has held that responsible position, earning the title of "the Greatest American Condenser," able to put, what is often necessary to be done in a paper of the size of the Sun, the meat of a column of matter into a paragraph, or a paragraph into a line. Both Mr. Cummings and Dr. Wood came from the Tribune, when that journal was a newspaper.[11]

The Herald, so far as news is concerned, stands quite on a par if not a little ahead of the Sun. Its weekly expenses in this department, are enormous, but it is edited with poor judgment and taste, and without that care and circumspection that is exercised by its more compact and compressed neighbor, and having more space to swing around in, very frequently "slops over." Most of the men holding responsible positions on the Herald, have been connected with it, a long number of years, but they are not at all known outside of the profession, the newspaper entirely swallowing up their personality. Dr. Thos. B. Connery, the Editor, is a plodding, industrious man, always to be relied on, but never brilliant and never

7—I think those old Athenian gossips who gathered about the baths, were the originals of the modern reporter, and if all that we read of them is true, what news editors they would have made.

8—The eagerness for exclusive news is as marked now, as when each journal had to depend upon itself for all it printed and when a paper has it, as last summer the Herald and Times printed the only accounts next morning of the attempted assassination of Washington Nathan, or the World recently, alone announced the removal of Police Commissioner Smith by Governor Robinson, it is referred to in the profession as a "beat," or according to the degree of its importance, as a "big beat." But in my observation, the enterprise or whatever you may call it, does not affect the public, however much it may excite those attached to rival publications. An habitual reader of the Herald will not buy a Times or Sun to get any exclusive intelligence, for he knows very well that on the next morning his own paper will contain all that he cares to know of the matter.

9—The ability to know news when he sees it, is another marked quality in a good editor. "He has a nose for news," is a common expression and formulates the notion as though it described a sense.

10—I say this, because you will find that the first newspaper a journalist always looks for, in the morning, is the Sun; where it is on file, it is always the most worn, and in the street cars or stages, or where there is a morning crowd, ten copies of it will be seen to one of any other newspaper.

11—The Tribune by rights ought to-day to be the greatest newspaper in point of influence and circulation in the country; it has a glorious past and a name by which to conjure. It could have a personal following perfectly enormous if it was in the hands of competent journalists, who could make it live up to its old traditions.

moving in any direction as to the policy or tone of the paper, by his own volition. "Jim" Bennett[12] controls his own property. Even the most competent man, under such circumstances, is blurred and dimmed and cannot do his best work, not feeling sure that it will suit the man who employs him. "Billy" Meaghan is just now[13] the City Editor, and he is one of the very best in the profession. William F. Smythe, the Night Editor, has held the position for many years, and there are George Williams, John Wilson, Joe Elliott, Dr. Wallis, Steve Hayes, and others, in responsible positions, who, although their work is seen every morning by thousands of persons, are no more known outside the office than is a stoker on an ocean steamer known beyond his vessel.

The Tribune is recovering somewhat from the terrible set back it got in the liberal movement, although it is esteemed doubtful if it can ever again assume the position it once held, at least under the control of so inefficient a man as Whitelaw Reid. It is not generally known, but it is a fact, that when he took the management, or very soon thereafter, the circulation of the weekly fell from 140,000 to less than 60,000, and the daily to less than 15,000. It was at a very low ebb until the deciphering of the cipher telegrams last year gave it a fictitious and temporary boost into favor.[14] John R. G. Hassard is the Managing Editor, William G. Shanks is the City Editor, and George W. Pearce[15] the Night Editor. It is owing to the good taste and judgment of Mr. Pearce that the Tribune is the handsomest looking paper printed in New York. His manner of arranging its news matter is very attractive and convenient. He makes the very best use of the material furnished him. The Tribune prints no Sunday edition and therefore beginning the week a day behind, it seems as though it would never catch up with its neighbors.

The World, a great many persons think, prints the most readable morning newspaper in New York. It is like Scribner's Monthly in the fact, that the average reader can begin at the first column, read through to the end and not find a paragraph or an article that is uninteresting. It may not always be news, but it is good reading. William Henry Hurlburt, the Editor, is a ready and sometimes brilliant writer. He was the author of the notorious editorial in the Times concerning the "elbows of the Mincio," and the "Quadrilateral." Ex-Mayor A. Oakey Hall is the City Editor, and its Managing Editor is Montgomery Schuyler, both men of ability.

The Star is comparatively a new paper. As every one knows it is the especial organ and advocate of John Kelly and Tammany Hall. Most of its local news is obtained from the City Association, and it is conducted, in all respects on the most economical not to say penurious plan. Its Managing Editor is a young gentleman by the name of Sanderson, who, at least, possesses great industry and application, if no particular genius or brilliancy. Charles H. File is the City Editor. The Star has on its staff a strong card in A. C. Wheeler or Nym Crinkle, the most noted and probably the best theatrical critic in New York. He is a little erratic perhaps, but is always bright and readable.

I have left the Times, you see, for the last, for I am not alone in never having been able to understand the reason of its existence or where it belongs. There are a great many things in this world hard to account for. For instance, what good end is reached by the creation of such a person as Adirondack Murray or Anthony Comstock? Then there is the publishing house of Appleton & Co. I never think of them without thinking of a great monument in a graveyard. It just stands there and does nothing else. Of course some one must patronize them and no doubt they do, or there would be no such firm, but I have yet to see the use of anything they ever issued. Take the Encyclopedia, for instance. It is utterly valueless as a work of reference. I never yet, in searching for information, got one ray of light from it. As an example, when Gen. Custer was massacred, it became my duty to say something of him and his career. I looked the Encyclopedia through, but found not one line in regard to him, although he had for years been filling a more than ordinarily conspicuous space in the public eye. It was the fourth or fifth time that the book had served me in a similar

12—Mr. Bennett is more generally spoken of thus, than by any more distant appellation.

13—I say "just now," because it would be difficult to prophesy what whim or caprice might induce Mr. Bennett to place another man to-morrow at the city desk and send Meaghan back into the reportorial ranks. He shakes things up in a lively manner sometimes when he is in the mood.

14—Of course it was only the political value of the ciphers that gave them any prominence, for as puzzles they were exceedingly simple. The St. Nicholas Monthly for girls and boys, has, in each issue, puzzles much more difficult of solution.

15—Also one of the Editors of the Sunday Times and Messenger.

manner, and I would sooner rely upon a last year's almanac for information, than Appleton's New American Encyclopedia. I would rather have a full set of Harper's Magazines, in fact, than all of the Encyclopedias in existence.

Then there are Lester Wallack and Wallack's Theatre. Pretence and veneering, a shabby gentility that has not even the poor foundation of blood, are as conspicuous in him and it as they are in an old hair cloth sofa that has done duty in the public room of an alms house for a century. And it is all veneering too. You can have some little respect for a piece of furniture even if it is veneered, if under the veneering you are sure there is some solid pine or white wood, but when you know it is all veneering! Pah! Lester Wallack an actor! His father knew better when he told him to the contrary. He can not and never could act any more than a broomstick which, no matter how fine may be the clothes, you hang upon it, is a broomstick still. The judgment of the country is correct on this pretentious old man, whose vanity has been the only quality he ever possessed. Last summer he started with a great flourish of trumpets to have a triumphant march across the country to San Francisco. He was accurately measured before he got beyond the Lakes and hastened home like a hen caught in a heavy shower of rain. It is to a strange class of persons that he caters. They must be shoddy and pretentious like himself. The leading actors and actresses that he employs, never meet with success elsewhere. They, no more than he, become favorites with the honest, fair-spoken, open-hearted people, who can see through shams at a glance, and who, thank Heaven! form the large majority of American citizens. Where else could Mrs. Hoey, who had about as much dramatic sense and intuition as a trying-on woman in a dressmaker's shop, have played and held any place at all, except with Lester Wallack? Or Mary Gannon, or Madeline Henriques, or Charles Walcot, or Henry Becket, or John Gilbert, or a score of others, who, among real actors are entirely out of place? It makes you think that something is all wrong in the "eternal fitness of things," to see so much assumed on such a frail basis and that, not of merit.

It is very much so with the Times. It was started out of pure spite and pique, two of the most contemptible feelings on which to found an action; when there was no more need or field for a new paper than there was for another moon. It has thriven only when its parent stalk the Tribune[16] has declined, and declines when that paper thrives. Had the Tribune fallen into more competent hands, the Times would, more than likely, not now be in existence. It was at so low an ebb just before the Tweed ring exposure, that it could hardly have lived six months longer, but the possession of the secrets of the greatest fraud this country ever knew, set it upon a highway that led to a financial success. George W. Jones is the Editor as well as virtual owner of the Times. His chief in the Editorial Department, is John W. Foord, who is a hard working, pains-taking man, and shines especially in after dinner speeches, when he gets a chance at one. John Reid is the Managing Editor. What qualifications he possesses for the position may be observable to the habitual reader of the journal, but the inextricable confusion in which its pages are usually made up is bewildering to an unaccustomed eye, and does not speak very highly for the mechanical ability of some one. Henry Lowenthal is the City Editor. He recently took the position held by the late Charles H. Pulham, one of the princes of the profession. Dithmar is the Night Editor. When to these names is added that of Richard Grant White, whose inane and vapid attacks on the English language are conceived with about the same skill and intelligence as marked the advance of King Cetywayo on English soldiers, the question as to the necessity for the publication of the Times becomes an exceedingly earnest and pertinent one.

I have taken no account of the policies or politics of the journals I have named. I have regarded them only as newspapers. In no other way is the business character of journalism, as it affects the great mass of those who follow it, more clearly shown; in no other way are its purely bread and butter getting qualities more definitely set forth, than in the fact, that whatever party lines the paper itself may adhere to, those working upon it are by no means bound by them. Some of the strongest Republicans in the city are attached to Democratic organs and vice versa. I know two or three members of the Tammany Society who earn their daily bread on the bitterest anti-Tammany sheets, and some good Republicans who never put President before Mr. Hayes's name when they write it.

16—The truth is, there is not room or field enough in New York for two first-class morning dailies of the same partisan leanings. Either, one of them will suffer and struggle; a constant drain on its friends and of benefit to no one, or both will live sickly and puny lives, accomplishing no good end.

